# Immunological and Clinical Responses to Vaccinations among Adults Living with HIV

**DOI:** 10.3390/life14050540

**Published:** 2024-04-24

**Authors:** Carlo Bieńkowski, Zuzanna Żak, Filip Fijołek, Martyna Cholewik, Maciej Stępień, Agata Skrzat-Klapaczyńska, Justyna D. Kowalska

**Affiliations:** 1 Hospital for Infectious Diseases in Warsaw, 01-201 Warsaw, Poland; filip.fijolek@gmail.com (F.F.); agata.skrzatasw@gmail.com (A.S.-K.); jdkowalska@gmail.com (J.D.K.); 2 Department of Adults’ Infectious Diseases, Medical University of Warsaw, 01-201 Warsaw, Poland; 3 Department of Internal Medicine, Endocrinology, and Diabetes, Medical University of Warsaw, 01-201 Warsaw, Poland; zuzanna.maria.zak@gmail.com; 4 Student’s Scientific Group at the Department of Adults’ Infectious Diseases, Medical University of Warsaw, 01-201 Warsaw, Poland; martynacholewik@gmail.com (M.C.); maciej.stepien98@gmail.com (M.S.)

**Keywords:** HIV, vaccinations, prevention

## Abstract

People living with human immunodeficiency virus (HIV) are at higher risk of morbidity and mortality due to vaccine-preventable diseases. At the same time, they are less likely to respond to vaccinations, and might have a higher rate of vaccine adverse event and faster waning of protective effect. International and national guidelines emphasize the importance of vaccinating people living with HIV against respiratory system disease pathogens including seasonal influenza, *Streptococcus pneumoniae*, and COVID-19, as well as against sexually transmitted infections, i.e., Hepatitis A and B (HAV, HBV) and human papillomavirus (HPV). This narrative review aims to provide a comprehensive examination of the current knowledge regarding the immune and clinical responses elicited by vaccinations in the older adult population living with HIV.

## 1. Introduction

People living with human immunodeficiency virus (HIV) are at higher risk of morbidity and mortality due to vaccine-preventable diseases, such as hepatitis B virus (HBV), severe acute respiratory syndrome coronavirus 2 (SARS-CoV-2), or pneumococcal infections [1,2,3,4]. At the same time, they are less likely to respond to vaccinations, and might have a higher rate of vaccine adverse event (VAE) and faster waning of protective effect [5]. Therefore, multiple restrictions apply to the HIV-positive population in terms of a choice of vaccine, immunization scheme, and optimal timing of vaccination, which may lead to vaccinations being omitted or neglected in this population [5,6,7].

Furthermore, the shared modes of transmission between HIV and other pathogens, such as hepatitis A and B, significantly increase the risk of infections among people living with HIV [7,8]. 

The introduction of highly effective antiretroviral treatment (HAART) resulted in a significant increase in life length in the HIV-positive population [9,10]. Therefore, with an aging population, immunosenescence also became an issue in people living with HIV [9]. The influence of immunosenescence on vaccination extends beyond its impact on antibody responses, encompassing alterations in T-cell-mediated responses that occur with aging and significantly affect the effectiveness of vaccination. Additionally, age-related modifications in the innate immune system can have significant implications for antigen presentation and the initiation of adaptive immune responses [11]. Notably, a persistent low-level inflammatory condition known as inflammaging has been demonstrated to hinder immune responses to vaccination, and there is potential for pharmacological approaches targeting baseline inflammation to enhance vaccine efficacy [11]. However, current strategies aimed at enhancing immunogenicity in the elderly have predominantly concentrated on utilizing adjuvants to stimulate localized inflammation [9,12].

International and national guidelines emphasize the importance of vaccinating people living with HIV against respiratory system disease pathogens including seasonal influenza, *Streptococcus pneumoniae*, and COVID-19, as well as against sexually transmitted infections, i.e., Hepatitis A and B (HAV, HBV) and human papillomavirus (HPV) [13,14]. 

Despite these recommendations, vaccination rates among people living with HIV remain consistently low, even lower than in the general population, raising concerns about the potential impact on herd immunity within this patient group [5,15].

This personal narrative review aims to provide a comprehensive examination of the current knowledge regarding the immune and clinical responses elicited by vaccinations in the adult population living with HIV. The goal is to contribute to a deeper understanding of vaccination efficacy in individuals within the context of HIV infection.

## 2. Vaccinations against Respiratory Pathogens

### 2.1. Vaccinations against Influenza

Tremblay et al. assessed a split-virion inactivated AS03-adjuvanted 2009 pandemic influenza A (H1N1) vaccine in 84 persons living with HIV [16]. Seroconversion and seroprotection rates were 67% and 70.2%, respectively, with no significant age-related differences. Microneutralization was more sensitive than hemagglutination inhibition assay, and it detected seroconversion in 19 more patients (56/84 vs. 37/84, *p* = 0.003). Titer levels for the same persons were higher when using microneutralization. Reported side effects were mainly mild or moderate, including local pain, fatigue, myalgia, and headache [16]. Fabbiani et al. evaluated an influenza A (H1N1)v MF59-adjuvanted vaccine in 41 HIV-infected individuals [17]. CD4+ cell counts influenced vaccine response, with higher counts (>200 cells/mm^3^) associated with better responses, both seroprotection and seroconversion. Post-vaccination response was measured via the geometric mean antibody titers which were significantly increased in patients with CD4+ >200 cells/mm^3^ at baseline (*p* < 0.001). Seroconversion and seroprotection rates were 61% and 78%, respectively. Responders with CD4+ >200 cells/mm^3^ showed better outcomes compared to nonresponders and those with CD4+ <200 cells/mm^3^ [17]. Durando et al. compared MF59-adjuvanted and nonadjuvanted influenza vaccines in healthy and HIV-1-infected adults [18]. The adjuvanted vaccine showed better immunogenicity, with seroprotection rates ranging from 61% to 82%. Adverse effects were mild, and HIV-1 infection did not influence plasma viral load or CD4+ cell counts [18]. Kelly et al. used a split vaccine with AS03 adjuvant in 81 HIV-infected individuals [19]. Responders had higher CD4+ cell counts (618, IQR 389–835 versus 446, IQR 314–582, *p* = 0.0157), and individuals with preexisting antibodies showed better outcomes (*p* = 0.00001), considering certain baseline mean fluorescence intensity as a positive response to vaccine. Vaccine administration was associated with a transient loss of circulating CD4+ T-cells, more pronounced in responders [19]. Biselli et al. studied antibody response to an H3N2 A/Shangai 16/89 strain in 13 HIV-infected individuals [20]. Baseline antibody levels were lower than in healthy controls; both HIV-infected and healthy elderly individuals showed significant increases in specific antibodies after vaccination [20]. Cagigi et al. investigated the role of activation-induced deaminase (AID) expression in predicting antibody response to an AS03-adjuvanted A(H1N1)pdm09 vaccine in 54 HIV-1-infected individuals [21]. AID expression correlated with antibody response, and individuals with higher AID expression maintained protective antibody levels for a more extended period. Protective levels of antibodies were still present after 6 months from vaccination (*p* = 0.04). [21]. Bickel et al. studied the seroconversion rate after a split-virion, inactivated, adjuvanted pandemic H1N1 influenza vaccine in 135 HIV-1-infected patients [22]. Nonresponders had lower nadir CD4+ cell counts, longer HIV infection duration, and higher baseline antibody titers. Adverse effects were mostly mild, with local reactions, joint pain, and fatigue reported [22]. Manuel et al. explored the immunogenicity of the influenza A H1N1/09 AS03-adjuvanted vaccine in solid-organ transplant recipients, HIV-infected individuals, and healthy controls [23]. HIV-infected individuals achieved similar responses to healthy controls, with seroconversion rates of 77% at day 21 and 87% at day 49 [23]. Yamanaka et al. investigated a split-virus A/California/07/2009 H1N1 vaccine with ASO3 adjuvant in 81 HIV-infected individuals [24]. Preexisting influenza antibodies and higher CD4+ cell counts were associated with better vaccine responses to each of the three vaccine components measured by the hemagglutinin inhibition assay. Vaccine administration was associated with a minor, transient loss of circulating CD4+ T-cells [24]. Yamanaka et el. investigated the efficacy and immunogenicity to a trivalent influenza subunit vaccine in 262 HIV-1-infected individuals. A total of 66 HIV-infected patients in this study did not receive the vaccine. Among vaccine recipients, median age was 41 (range 20–78) and their median CD4+ cell count was 380/mm^3^. A total of 75.2% of them were treated with antiretroviral drugs. Individuals who did not receive the vaccine were younger (median age 40, range 20–61) and had fewer median CD4+ cells count (374). In total, 72.3% of them were on antiretroviral therapy. Influenza was more frequent among unvaccinated persons (incidence = 6.1% (95% confidence CI: 4–10%) in vaccinated vs. 21.2% (CI: 13–35%) in nonvaccinated persons, *p* < 0.001; relative risk = 0.29 (CI: 0.14–0.55)). Among baseline antibody-negative patients, anti-H1 and anti-H3 antibody responses to the vaccination were significant in those patients with a CD4+ cell count of more than 200 cells/mm^3^ compared with those with a CD4+ cell count of less than 200 cells/mm^3^ (*p* < 0.05). On the contrary, in patients with present antibodies at baseline, good antibody responses were observed irrespective of CD4+ cell counts. Specific CD4+ responses correlated with HIV viral load, especially in patients treated with combined antiretroviral therapy compared with those who were treatment-naive (*p* < 0.01). The vaccine response was not affected by viral plasma load. O’Brien et al. showed that HIV-1 replication increased post influenza vaccination [25]. The study suggested a potential impact on viral load, contrasting with other studies [25]. George et al. studied immune response to trivalent inactivated influenza vaccine in young and old HIV-infected and healthy individuals [26]. Seroprotection rates were lower in HIV-infected individuals (55–93% versus 75–100%), with an inverse correlation between antibody titers and age [26]. In a follow-up study, George et al. investigated factors contributing to impaired vaccine response in HIV-infected persons [27]. CD11b+ inflammatory monocytes increased with age, negatively impacting antibody responses [27]. Iorio et al. studied antibody response and HIV-1 viral load after influenza vaccination [28]. Adjuvanted vaccine recipients had higher antibody titers, and vaccine administration did not influence CD4+ cell counts or viral replication [28]. Pariani et al. researched the coadministration of a monovalent AS03-adjuvanted 2009 A(H1N1) pandemic influenza vaccine and a seasonal trivalent vaccine in HIV-infected and healthy subjects [29]. HIV-infected individuals reached similar responses to healthy controls. The percentage of individuals with protective antibody titers for each antigen significantly increased in both of these groups [29]. Launay et al. investigated the AS03A-adjuvanted H1N1v vaccine in 306 HIV-infected adults [30]. Adjuvanted vaccine recipients showed higher immune responses than nonadjuvanted vaccine recipients considering seroconversion rates and factor increases in geometric mean titers (*p* < 0.001). No impact on CD4+ cell counts or viral load was observed [30]. Powell et al. assessed T-cell responses to influenza A/California/7/2009 vaccine in HIV-infected and healthy individuals [31]. Reduced CD4+ and CD8+ cell responses were observed in people living with HIV, indicating potential challenges in cell-mediated immunity [31]. Pallikkuth et al. investigated mechanisms influencing antibody response to nonadjuvanted 2009 H1N1 influenza vaccine in HIV-infected individuals [32]. Responders upregulated IL-21R on B cells and increased serum IL-21 levels, while nonresponders lacked these immunologic responses [32]. In a follow-up study, Pallikkuth et al. examined innate immune factors affecting B cell function in nonresponders to H1N1/09 influenza vaccine [33]. Nonresponders had lower frequencies of BAFF-R and TACI expressing memory B cells, indicating impaired responses [33]. In another study, Pallikkuth et al. investigated the effect of age and HIV infection on influenza vaccine responses [34]. HIV-infected individuals showed lower vaccine responses, with significant differences in the young age group [34]. Moysi et al. explored tissue-resident immune cell dynamics after trivalent influenza immunization in HIV-infected individuals [35]. Follicular architecture alterations were observed in lymph nodes, with reductions in follicular helper T-cells after vaccination [35]. McKittrick et al. assessed the immunogenicity of a high-dose seasonal trivalent influenza vaccine in HIV-infected persons [36]. The high-dose group exhibited higher seroprotection rates across influenza strains, with similar adverse effects [36]. Chawansuntati et al. investigated cell-mediated responses to an influenza A/California/7/2009 vaccine in HIV-infected individuals [37]. Cytokine-producing and CD107a-expressing T-cells showed similar increases in both HIV-infected and healthy individuals, while memory T-cell responses were lower in HIV-infected persons [37].

In summary, these studies highlight the variability in influenza vaccine responses among HIV-infected individuals, with factors such as age, CD4+ cell counts, preexisting antibodies, and inflammatory monocytes influencing outcomes. Adjuvanted vaccines generally showed better immunogenicity, but challenges persist in achieving optimal responses in this population.

### 2.2. Vaccinations against Streptococcus pneumoniae

Current guidelines for HIV-infected patients recommend vaccination with a 15-valent or 20-valent pneumococcal vaccine (PCV15, PCV20), followed by a 23-valent pneumococcal polysaccharide vaccine (PPV23) [38]. A 7-valent and a 13-valent conjugated vaccine (PCV7, PCV13) have also been available in the past, with PPV23 being the subject of most studies on pneumococcal vaccination among HIV-positive patients [39,40,41,42,43,44,45,46,47,48,49,50,51,52,53,54,55].

No serious adverse reactions were reported after PCV and PPV23 vaccination of HIV-positive patients. The number of PCV13 doses administered (1 vs. 2) and the type of vaccine (PCV7 vs. PPV23) did not affect the incidence of adverse reactions [49,51]. According to K. Slayter et al., side effects were more frequently (*p* < 0.005) reported by patients with a CD4 count below 200 cells/mm^3^ (12). The vaccines are considered safe and do not cause a decrease in CD4 count [39,40,41,50].

According to F. Lombardi et al. the two doses of PCV13 were as safe and well-tolerated as the single dose of PPV23 [45]. It has been observed that the response to PPV23 vaccination measured with antibody titers in patients with HIV is weaker than in the healthy population [40,44,52]. J. Ballet et al.’s study showed that patients with AIDS-related persistent generalized lymphadenopathy had lower mean increases after PPV23 vaccination for IgM (*p* < 0.002), IgA (*p* < 0.002), and IgG2 (*p* < 0.05) serotypes [44]. According to N. French et al., one month after PPV23 vaccination, only HIV-seronegative control subjects showed significant rises in antibodies to serotype 19F; the mean IgG level in seropositive patients increased only from 21 mg/L (CI: 12–30) to 29 mg/L (CI: 17–41). In other serotypes, levels of capsule-specific IgG were also lower in HIV¬1-infected patients [52]. 

Nevertheless, among HIV patients, the incidence of pneumonia in vaccinees (PPV23) is lower than in nonvaccinees (2.1 vs. 21.8 per 1000, *p* = 0.007) [41]. In the study conducted by C. Hung et al., only one of 305 vaccinated individuals had pneumococcal pneumonia at the time of the observation, and that was an elderly person aged 76 [41]. 

P. Lesprit et al., in their survey, compared the administration of PPV23 alone with PCV7 with PPV23 boost. After 24 weeks, the boost strategy led to a higher level of specific IgG against six of seven polysaccharides (4, 9V, 14, 18C, 19F, and 23F) shared by the two vaccines, four of which were statistically significant (14 (*p* = 0.01), 18C (*p* < 0.01), 19F (*p* < 0.01), and 23F (*p* < 0.01)) [48]. Similar conclusions were reached by C. Rabian et al., who compared the proliferative response of lymphocytes to the diphtheria carrier protein CRM197 contained in PCVs. Responses 24 weeks after original vaccination were significantly higher in the PCV and PPV group than in the PPV group (*p* < 0.001) [54]. According to F. Lombardi et al., both PCV13 and PPV23 showed comparable immunogenicity at the end of the follow-up (48 weeks). The exception was immunogenicity and seroprotection regarding serotype 3, where the percentage of responders was significantly lower in the PCV13 group [45]. A. Cheng et al. compared the efficacy of two doses of PCV7 vs. one-dose PCV7 protocol. At the end of the five-year follow-up, more HIV-infected participants in the two-dose group (61.8% vs. 76%, *p* = 0.026) had persistent serological responses [51].

According to D. Kvale et al., low IgG2 or high IgM levels at baseline were associated with low responses, whereas high IgG4 or low ß2 microglobulin levels were connected with good responses to PPV23 [43]. According to T. Johannesson et al., isotype-switched memory B cells were a predictor of the 3-, 4-, and 9-month IgG response to PCV7 [46]. J. Romaru et al. found that a CD4/CD8 ratio > 0.8 was significantly associated with a better global response by OPA [39]. 

Many studies have concluded that the level of CD4 count in vaccinated patients (PPV23, PCV13) does not affect the immune response [40,43,45]. J. Romaru et al. disagreed, noting that one month after vaccination (PCV13), HIV-positive patients with a CD4 count nadir below 200 cells/µL were less likely to have global protection (OR = 0.18 CI: 0.03–0.96, *p* = 0.04), according to opsonophagocytic assay (OPA) [46]. Moreover, according to K. Slayter et al., patients with CD4 counts <200 cells/mm^3^ were more likely to produce antibody responses 6 months (*p* = 0.04) and 12 months (*p* = 0.004) after vaccination (PPV23, PCV7) following immune reconstitution (12). 

Smokers (OR = 0.38 CI: 0.22–0.64, *p* = 0.0004) and those concurrently infected with HCV (OR = 0.25 CI: 0.12–0.54, *p* = 0.0004) were less likely to respond to vaccination (PCV7, PPV23), according to P. Lesprit et al. [48]. Moreover, in a study conducted by J. Romaru et al., a group of HIV-positive patients aged over 50 were less likely (OR = 0.15, CI: 0.03–0.70, *p* = 0.02) to be globally protected after PCV13 according to OPA results [39]. According to O. Sogaard et al., the addition of CPG 707 adjuvant, a toll-like receptor 9 (TLR9) agonist, increased the proportions of HIV-infected patients developing a high vaccine-specific IgG antibody response to PCV7 (10 months after vaccination (*p* < 0.001). However, the addition of CPG 7909 to PPV23 is not desired because it did not amplify the antibody response to non-PCV7 serotypes and increased the incidence of moderate to severe influenza-like reactions [55].

R. Offersen et al. showed that CPG 7909 did not affect antibody class switch but provoked an overall higher relative cytokine response, in particular Yh1-cytokine IFN-γ (*p* = 0.0047), the inflammatory markers IL-1ß (*p* = 0.0046) and IL-6 (*p* = 0.0051), the chemokine M1P-1ß (*p* = 0.0086), and IL-2 receptor (*p* = 0.0062) [47]. M. Deloria-Knoll et al. tried zinc and vitamin A supplementation during vaccination (PCV7) of HIV patients, but neither had any effect on antibody responses [42]. According to R. Ranieri et al., pneumococcal vaccination (PPV23) can be combined with the one against influenza, reducing the incidence in HIV-positive patients of both diseases. In that study, no cases of pneumonia were reported in the vaccinated, and there were significantly fewer cases of influenza (13.3% vs. 61.6%, *p* < 0.0001) [53].

In the studies presented here, the representation of people over 60 years of age is poor, with six of them having no such patients at all [40,44,47,50,52,55]. Moreover, in six of them, it is not distinguished whether this age group participated in the study [39,42,45,48,51,54]. People aged 60 and over were included in five surveys [41,43,46,49,53]. This is important because a study by D. Goldblatt et al. showed that in the HIV-negative population over 50 years old vaccinated with PCV7 or PPV23 serotype-specific IgG concentrations were typically 10% lower for each 10 years of increasing age [56]. For this reason, it is recommended to check the response to pneumococcal vaccination in HIV-positive patients over 60 years of age.

### 2.3. Vaccinations against COVID-19

Considering the onset of the COVID-19 pandemic in early 2020 and the major global threat, the development of a vaccine against SARS-CoV-2 became a new priority in vaccinology [57]. An increased risk of a severe clinical course of the disease was rapidly observed in the elderly and those with comorbidities, including patients living with HIV [58,59]. The first clinical trials on the effectiveness of the newly developed vaccines began to emerge, including in high-risk subpopulations [60].

Among these trials, the Sisonke study evaluated the efficacy of a single dose of the Ad26.COV2.S vaccine in healthcare workers during the COVID-19 pandemic, encompassing elderly and HIV-infected subpopulations [61]. The findings indicated comparable efficacy of the vaccine for COVID-19-related hospital admission and hospital admission requiring CCU or ICU treatment in both HIV-positive and HIV-uninfected healthcare workers [61]. Moreover, while healthcare workers with HIV were protected by the vaccine against COVID-19-related death, the risk was reduced compared to HIV-negative healthcare workers (65% (13 to 93) vs. 83% (72 to 97) in subgroup B). Although a larger percentage of participants were middle-aged (aged 40–60), the study also included older individuals. Interestingly, subgroup analyses of the elderly revealed a comparable effect of Ad26.COV2.S. Vaccine effectiveness against COVID-19-related hospital admission was 68% (59 to 76) in scheme B in subpopulations 18–49 years old vs. 67% (59 to 75) in subpopulations ≥ 50 years. The results were similar in the reduction in COVID-19-related deaths and were 70% (40 to 94) vs. 84% (72 to 93) in these subgroups [61].

Aberg et al. provided novel information on the mRNA vaccine’s effectiveness, the immune system’s response, and the effect of mRNA vaccines on virological control. Despite anti-RBD antibody titers being lower than those observed in healthy volunteers (156.1 IU/mL (95% CI 110.8–220.0), 2372.0 IU/mL (95% CI 2192.3–2566.4), and 1303.4 IU/mL (95% CI 1075.7–1579.2)) at three different timepoints, respectively, vs. 308.5 IU/mL (95% CI 206.6–460.6), 2815.6 IU/mL (95% CI 2677.9–2960.3), and 1896.5 IU/mL (95% CI 1611.4–2232.1), people living with HIV on antiretroviral therapy generated adequate anti-RBD antibody titers up to 4 months after receiving two doses of the SARS-CoV-2 mRNA vaccine [62]. Additionally, there were no significant differences in serological responses regardless of HIV viral load. Vaccine tolerability was also satisfactory, demonstrating the safety of COVID-19 mRNA vaccines [62]. Although the average age of the patients in this study was 54 years, the study included patients over 60 years of age [62].

Hensley et al. investigated the immunogenicity and reactogenicity of SARS-CoV-2 vaccinations in people living with HIV, considering both mRNA and viral vector vaccines [63]. Patients remained on effective cART; almost 98% had <50 copies/mL of HIV plasma RNA. The response rate after receiving an mRNA vaccine for HIV-infected patients was 93.6%, whereas all controls had an adequate response [63]. The poorer outcome after vaccination applied to all vaccines used in the study. In participants receiving mRNA vaccines, other factors that correlated negatively with vaccination efficacy, although less than having HIV infection (39.35% lower antibody concentration in PLWH compared to HIV- negative controls (0.607, 95% CI 0.508–0.725, *p* < 0.001), were male sex (23.05% (0.769, 95% CI 0.667–0.888)) and age > 65 years (35.47% (0.645, 95% CI 0.544–0.765)), both *p* < 0.001) [63]. When the HIV RNA level was above 50 copies/mL, a significant effect of lower antibodies was additionally identified (0.454, 95% CI 0.286–0.720, *p* = 0.001). Age, sex, and a detectable viral load did not influence the antibody levels. However, the use of the vector vaccine compared to mRNA vaccine correlated with 39.47% lower response in people living with HIV (0.605, 95% CI 0.387–0.945, *p* = 0.027), whereas the age, sex, and a detectable viral load did not influence the antibody levels. Regarding both vaccines, the most recent CD4+ T-cell count between 250 and 500 cells/L or higher was associated with the greatest positive impact on antibody levels (both *p* < 0.001) [63]. SARS-CoV-2 spike protein exposure was associated with an overall increase in T-cell responses (*p* = 0.002), including CD4+ and CD8+ T-cell activation as well as cytokine production. The SARS-CoV-2 vaccines proved to be well tolerated in people living with HIV, with no vaccine-related serious adverse events (SAEs) [63] (Table 1). 

In conclusion, people living with HIV typically exhibit a lower antibody response to SARS-CoV-2 vaccines compared to HIV-negative controls. Therefore, additional vaccinations need to be considered to compensate for this decreased antibody response, particularly among the elderly population. Additionally, mRNA vaccines should be preferentially selected for HIV-positive patients. The vaccines against COVID-19 are well tolerated in this subpopulation. 

## 3. Vaccinations against Sexually Transmitted Pathogens

### 3.1. Vaccinations against Hepatitis A

Acute HAV infections are typically self-limiting, and have been associated with a significantly higher risk of death among patients with chronic liver disease [64]. Vaccination is therefore recommended for any HIV-infected patient at an increased risk of HAV infection without anti-HAV antibodies and for those likely to develop severe liver disease [65].

Jimenez H. et al. investigated the immune response to HAV vaccination among HIV-infected patients in their study [64]. Only 53.5% of HIV-positive patients developed anti-HAV antibodies after vaccination. Those who responded positively to vaccination had a baseline higher CD4 lymphocyte count (446/mm^3^ vs. 362/mm^3^, *p* = 0.004), lower HIV RNA levels (475 copies/mL vs. 5615 copies/mL, *p* = 0.008), and a higher rate of HIV viral suppression (48% vs. 32%, *p* = 0.024) compared to those who did not develop anti-HAV antibodies. It is worth noting that the median age of the people included in the study was 41.8 years (range 18–58 years), so it is not possible to determine from this study what response would have occurred in people over 60 years of age [64].

In the study by Launay O et al., 95 HIV-1-infected patients aged 18–55 years were included [66]. Patients were randomly assigned to intramuscularly receive three doses at weeks 0, 4, and 24 (46 patients) or two doses at weeks 0 and 24 (49 patients). Seroconversion rates were observed at 4, 8, 24, 28, and 72 weeks after the first dose of the vaccine. When seroconversion was examined at week 24, the differences between the two groups were greatest, in favor of the group receiving three doses of vaccination (74.4% vs. 46.8%, *p* < 0.01) [66]. However, this is probably due to the fact that these patients had already received two doses compared to patients in the other group who had only received one. In contrast, at week 28, i.e., four weeks after the last doses were taken, seroconversion was observed in 88.4% of patients in the three-dose group whose samples were available and in 72.3% of the two-dose group [66]. At week 72, i.e., one year after the last dose, anti-HAV antibodies were detected in 85.7% of patients in the three-dose group and 69.8% of those in the two-dose group [66].

Kourkonti S. et al. also included only patients under 60 years of age [67]. It was noted that patients who received HAART treatment had higher geometric mean anti-HAV antibody titers than those who did not receive the treatment, but it should be noted that this difference was not significant (237 mIU/mL vs. 158 mIU/mL, *p* = 0.068). The baseline CD4+ cell count had no effect on anti-HAV antibody levels after vaccination [67]. It is noteworthy that there was a significant decrease in CD8 T-cell (1020/mm^3^ vs. 972.5/mm^3^, *p* = 0.002) and CD4 + CD8 + T-cell (3.5/mm^3^ vs. 2.0/mm^3^, *p* = 0.003) counts after vaccination in HAART-treated patients, whereas no such changes were observed in the group of patients not treated with HAART [67].

The study by Fritzsche C. et al. also involved patients under the age of 60 years [68]. In this study, one group of patients was vaccinated against HAV only (81.5% of individuals developed antibodies, and the response to vaccination was more frequent, with a better response in patients with a higher CD4 cell count (580/µL vs. 355/µL, *p* = 0.003) and CD4/CD8 ratio at vaccination (0.54 vs. 0.37, *p* = 0.032), while the other group was vaccinated against both HAV and HBV (79.2% developed antibodies and vaccination response was more common, with a better response to the vaccine in younger patients (37 years vs. 47 years, *p* = 0.043) and females (100% women vs. 73.8% men, *p* = 0.003. As this study shows, the type of vaccine (only against HAV or simultaneously against HAV and HBV) does not influence the vaccine response [68].

Cheng A. et al. assessed the persistence of anti-HAV antibodies in HIV-infected men who received two or three doses of vaccination [69]. The level was tested every 2 years for 5 years. It was shown that at 5 years, geometric mean anti-HAV IgG antibody levels were more often ≥20 mIU/mL in the three-dose group than in the two-dose group (OR = 3.36, *p* = 0.03), which may encourage HIV-infected patients to be vaccinated with three doses of HAV vaccination [69].

None of the studies that analyzed the response of HIV-positive patients to HAV vaccination considered patients over 60 years of age; this is a significantly underrepresented group, but it is reasonable to expect that the results in this group could be similar.

### 3.2. Vaccinations against Hepatitis B

Coinfection with HIV and HBV is associated with a nearly 15-fold (*p* < 0.001) increase in mortality compared to HBV mono-infection [70].

Laksananun N. et al. demonstrated that the administration of one dose of the HBV vaccine to HIV-infected patients who also have anti-HBc antibodies resulted in an immune response in only one in three patients [71]. They also showed that the administration of three or four doses of the vaccine (patients in both groups were 45.8 ± 13.5 years and 46.6 ± 11.0 years, respectively) against HBV was effective and should be recommended in HIV-infected patients. Antibody levels at week 28 were significantly higher in patients vaccinated with the four-dose regimen (209.8 mIU/mL vs. 63.8 mIU/mL, *p* = 0.030) [71]. The results of the study were not correlated with age, so it may be reasonable to use the extended four-dose HBV vaccination schedule in patients over 60 years of age [71].

Kalinowska-Nowak et al., in their study involving a group of people aged 20–64 years, also showed that an increase in the vaccine dose or the number of doses could influence a better immune response in HIV-infected patients over 60 years; anti-HBs antibodies >10 IU/L after the first, second, and third additional doses of the vaccine had results of 79.7%, 87.1% and 90.7%, respectively [72].

Chaiwarith R. et al., in a study on HIV-infected patients over 18 years of age, showed that the use of standard doses (20 μg at months 0, 1, and 6) of the HBV vaccine in these patients may be associated with an insufficient immune response [73]. Four double doses (40 μg at months 0, 1, 2, 6) may be recommended (anti-HBs antibodies ≥ 10 mIU/mL in 57.1% of patients vaccinated with the standard dose vs. 80.5% of patients vaccinated with four double doses, *p* = 0.033) [73]. Chawansuntati K. et al., in their study on an HIV-infected population, showed that the use of increased doses and/or frequencies, compared to a healthy population, may be beneficial for this population [74].

According to the study by Bailey C. et al., vaccination response is strongly associated with a CD4 count > 350/mm^3^ (*p* = 0.006) and an undetectable viral load (*p* = 0.001) at the time of vaccination. Only two patients were over 60 years of age, and both of these patients had a positive immune response after vaccination [75]. Lack of response to the vaccine was most significantly associated with a high HIV viral load of >100 000 copies/mL (3 responders vs. 13 nonresponders, *p* = 0.0009). No association was detected with age, race, weight, tobacco use, HAART use, or HCV coinfection [75].

Veiga A. et al. also concluded that a higher CD4+ total T-cell count (452/mm^3^ vs. 359/mm^3^, *p* = 0.034)) and a lower viral load (2.86log10 vs. 3.63log10, *p* = 0.034) on the day of vaccination positively correlated with a positive immune response after HAV vaccination. In a group of 19 patients with an age confidence interval of 21–60 years and a CD4+ cell count ≥500/mm^3^, antibody levels were high after vaccination [76]. This may indicate a good immune response in patients older than 60 years with a baseline CD4+ cell count of ≥450/mm^3^ [76].

Kalinowska-Nowak et al. showed that the higher the CD4 lymphocyte count (*p* = 0.015) and lower the viral load (*p* = 0.015), the better the immune response to HBV vaccination [72].

Herrero-Fernández I. et al. concluded that HIV-infected patients treated with cART containing maraviroc show higher antibody titers after HBV vaccination (1000 mIU/mL vs. 720 mIU/mL, *p* = 0.048) [77]. The magnitude of response in patients on maraviroc-containing therapy who had previously been vaccinated against HBV and received simultaneous vaccination against HAV was significantly higher in patients under 50 years of age (*p* = 0.009), so it can be assumed that there is no such relationship in patients over 60 years of age, but additional studies are needed. Furthermore, it was shown that one year after the first dose, antibody titers were higher in female patients who had been concomitantly vaccinated against HAV and who had been previously vaccinated against HBV [77]. 

Miller E. et al. studied the immune response to HBV vaccination in patients with congenital coagulation disorders (coagulation factor VIII or IX levels less than 20 units/dL) [78]. Fifteen patients aged in the range of 11–72 years having anti-HIV antibodies were vaccinated against HBV, and antibody levels were measured. Eight of these patients were also anti-HBc positive, indicating previous HBV infection. A significant proportion of anti-HIV-positive and anti-HBc-positive or -negative patients did not respond to vaccination, while anti-HIV-negative/anti-HBc-positive patients showed such response [78]. This may indicate a negative effect of being HIV-infected on the immune response to HBV vaccination. It has also been demonstrated that subcutaneous vaccination is more immunogenic in patients with congenital coagulation disorders than intradermal vaccination (90% vs. 56%, *p* = 0.016), and that a doubling of the vaccination dose may be necessary in HIV-infected patients [78]. 

In a prospective study by Morsica G. et al., 25 HIV-infected patients in the age range of 45–54 years were vaccinated against HBV [79]. All patients had anti-HBc antibodies, and 14 (56%) additionally had anti-HCV antibodies. It was shown that the longer the duration of HIV infection (27 years vs. 12 years, *p* = 0.044) and the presence of anti-HCV antibodies (80% of responders vs. 29% of nonresponders, *p* = 0.023) had a positive effect on the immune response, whereas CD4+ cell count or age had no such relationship [79]. Therefore, it can be thought that in patients over 60 years of age who have a long history of HIV infection and additionally have anti-HCV antibodies, the immune response to vaccination may be satisfactory [79]. 

Patients who had an anti-HBs titer of ≥10 IU/L 28 weeks after vaccination were included in the study by Lopes V.B. et al. These antibody titers were measured annually for 5 years [80]. Patients with decreasing anti-HBs titers < 10 IU/I were defined as patients with a transient response and those with persistent anti-HBs titers ≥ 10 IU/I as patients with a long-term response. Persistence of effective anti-HBs titers was significantly associated with higher antibody titers after primary vaccination. There were more patients aged >60 years in the transient response group (21.5–64.6 years vs. 32.3–45.6 years in the long-term response group), but this difference was not significant [80]. Nevertheless, this may indicate a weaker immune response after HBV vaccination in older people.

Viard J-P. et al., in their study on 339 HIV-infected patients with a median age of 43 years, investigated the presence of a positive correlation between vitamin D and the immune response to HBV vaccination [81]. However, such a correlation was shown not to exist (*p* = 0.09) [81]. 

Cooper C. et al. evaluated the effect of the ologideoxynucleotide adjuvant CPG 7909 (contains immunostimulatory CpG motifs) [82]. Inoculations with Engerix B with the above adjuvant were well tolerated locally and systemically, and the addition of the CPG 7909 adjuvant provided rapid, higher, and more durable seroprotection in HIV-infected individuals [82]. Antibody titers of at least 10 mIU/mL after six weeks were detected in 89%, after eight weeks in 89%, and after 12 months in 100% of those receiving CPG 7909 versus 53%, 42%, and 63% of those in the control group, respectively (*p* = 0.029, 0.005, and 0.008). Therefore, one may consider the use of this adjuvant in populations poorly responsive to the HBV vaccine, including HIV-infected patients over 60 years of age.

Overton E.T. et al., in their study, tested whether the use of GM-CSF (granulocyte-macrophage colony-stimulating factor) as an adjuvant influences a better immune response in HIV-positive patients [83]. The study included mainly young patients, whose median age was 41 years. The results show that HBV vaccine with GM-CSF as an adjuvant does not improve the development of HBsAb antibody titers. In fact, 4 weeks after vaccination, the observed response rate was slightly higher in the group vaccinated without the adjuvant compared to the group that was vaccinated with the vaccine containing GM-CSF (65% vs. 52%, not statistically significant) [83]. 

In a study by Anthony DD. et al. on a group of subjects with a median age of 47 years, it was shown that the addition of GM-CSF to the HBV vaccine may be associated with a weaker immune response to the vaccine, so it should also not be recommended in patients older than 60 years [84]. 

Chakvetadze C. et al. assessed the immune response after vaccination against HBV in 40 HIV-infected patients with a median age of 40 years (29–52) and found that there were no factors that were predictors of the immune response [85]. In this study, 74% of patients achieved a positive immune response after the administration of 3–6 doses of the vaccine, so it can be assumed that a similar response would be observed in patients over 60 years of age [85].

The group of HIV-infected patients over 60 years of age is highly underrepresented in research. New research is needed, and most conclusions can be drawn indirectly from work carried out on other age groups.

### 3.3. Vaccinations against Human Papilloma Virus (HPV)

The first reported study of a therapeutic vaccine directed against human papillomavirus (HPV) antigens to treat high-grade anal intraepithelial neoplasia (HG-AIN) in HIV-positive individuals was conducted in 2006 [86]. Since HG-AIN is more prevalent in HIV-positive patients, it seemed crucial to investigate the effectiveness of HPV vaccination in this population, while it was anticipated to be a therapeutic option. The vaccine used in this phase I/II trial consisted of a fusion of HPV 16 E7 protein and the Mycobacterium bovis heat shock protein 65, and it was well tolerated by the study participants [86]. The average age was 47.5 years. The participants’ exact ages were not specified, but it cannot be ruled out that those over 60 participated. Unfortunately, there was a decrease in CD4 count (*p* = 0.05) and CD8 (*p* = 0.04) count after vaccination, with CD4/CD8 ratio remaining stable (*p* = 0.23). Regardless of the vaccine dose used, there was no significant rise in viral load over the course of the 24 weeks of the study [86]. Nevertheless, some patients obtained complete or partial responses, which prompted further research into this topic.

In the study by Anderson et al., the median age of the participants was 47 years (18–60 years range). Administration of the HPV-16 E6E7 ISCOMATRIX vaccine produced an antibody response in almost all study vaccine recipients [87]. After the third vaccination, the humoral response persisted for a long time, with antibodies still present 24 to 26 weeks later. The Th1-type IFN-γ response rate was 71.4% (20 of 28) and IFN-γ geometric mean concentrations were significantly higher in the actively treated subpopulations vs. the placebo group after the first (*p* = 0.0384), second (*p* = 0.0177), and third vaccination (*p* = 0.0032). For the entire course of the study, mean CD4 cell counts in all treatment groups remained relatively unchanged (ranges between 486 and 759 cells/μL). Transiently detectable viral loads were reported in some patients. There were no serious adverse events reported [87]. Contrary to the previously described study, the examined vaccine did not result in any clinical response; however, there were patients included whose disease was caused by other types of HPV than HPV-16 [87]. 

The efficacy of the HPV-16/18 vaccine (Cervarix) was evaluated in a population of young women (aged 18–25 years) in South Africa. Interestingly, in this case, HIV testing was conducted during screening, so some of the patients were unaware of their HIV status [88]. The disease had to be World Health Organization (WHO) Clinical Stage 1. Only two participants were on antiretroviral therapy at baseline, so viral loads were unquestionably higher than in previous studies. During the study, some participants began receiving antiretroviral therapy. Both HIV-positive and HIV-negative women had satisfactory and comparable immunological responses [88]. All patients were seropositive for HPV-16 and 18 after the second vaccination dose, and this continued until the end of the observation. Anti-HPV-16 and 18 antibodies peaked at month 7, after receiving all doses. Geometric mean antibody titers (GMT) were 3558.2 (95% CI: 2723.6; 4648.6) EL.U/mL in the HIV-infected patients and 8168.8 (95% CI: 6341.0; 10,523.5) EL.U/mL in the HIV-negative subpopulation. Corresponding anti-HPV-18 antibody GMTs at the same timepoint were 1945.8 (95% CI: 1451.4; 2608.6) and 3703.0 (95% CI: 2502.5; 5479.4) EL.U/mL. Treatment tolerability was satisfactory despite the reported AEs and did not depend on HIV status. SAEs that happened during the study were determined to be unrelated. The CD4 T-cell count and viral loads remained stable, indicating that HPV vaccination had no negative impact on the management of HIV infection [88]. Unfortunately, the study did not include patients over 60 years of age. 

Money et al. assessed the efficacy of a quadrivalent HPV 6,11,16,18 (qHPV) vaccine in HIV-positive women aged 15–66 years [89]. Almost all participants in the per-protocol population experienced seroconversion 98.9% (96.2–99.9%; *n* = 186), which was consistent with population data from HIV-negative individuals. The baseline HIV viral load was the only variable that could be linked to a worse immune response. The GMTs ratio (95% CI) for patients with suppressed VL (<40 copies/mL) vs. those with ≥40 copies/mL was 2.02 (1.29, 3.16), 2.19 (1.60, 3.00), 1.74 (1.17, 2.59), and 3.05 (1.94, 4.80) for HPV types 6, 11, 16, and 18, respectively. Neither the mean CD4 count nor the number of viral copies was impacted negatively by the HPV vaccine [89]. The qHPV vaccine was safe and well tolerated by the participants. Only one VAE was thought to be possibly connected to the vaccine. This is another study that demonstrated improved HPV vaccination effectiveness with suppressed viral loads [89]. To maximize the antibody response, vaccination timing should be considered once virologic suppression has been achieved. Most of the women in the study were seronegative for at least one type of virus at baseline, even though many of them were older than the typical HPV vaccination age [89]. 

The studies on therapeutic HPV vaccines targeting high-grade anal intraepithelial neoplasia (HG-AIN) in HIV-positive individuals have shown promising outcomes despite some variations in vaccine formulations. While the vaccines were generally well tolerated, they elicited variable immune responses and clinical outcomes (Table 1). Notably, some patients exhibited complete or partial responses, indicating the potential of HPV vaccination as a therapeutic option in this population. However, further research is warranted to optimize vaccine efficacy, considering factors such as age, HIV status, and viral load suppression.

## 4. Limitations

This paper being a narrative review has its limitations. The keywords used for comprehensive research of the PubMed data base were “HIV”, and “Immunological response”, and “vaccination”, and “adults”, and “elderly”. We may have missed any additional and relevant key word for literature search, potentially leading to the omission of pertinent studies. In addition, due to limited search results, our presumptive objective may have not be fully implemented. However, our results also provide some insight in the topic, showing limitations on the data and the necessity for further research in this regard. Future studies should aim to address these limitations, employing more exhaustive search strategies and robust methodologies to provide a more comprehensive understanding of the effectiveness and safety of vaccinations in HIV-infected individuals.

## 5. Conclusions

In summary, influenza vaccine responses in HIV-infected individuals are influenced by various factors (age, CD4+ cell counts, preexisting antibodies, and inflammatory monocytes), and adjuvanted vaccines demonstrate improved immunogenicity (Table 1). Pneumococcal vaccines, particularly PCV15 or PCV20 followed by PPV23, are recommended, but weaker responses to PPV23 are noted. COVID-19 vaccines, including Ad26.COV2.S and mRNA vaccines, show effectiveness and safety in people with HIV, with mRNA vaccines recommended, especially for older individuals (Table 1). Hepatitis A and B vaccination responses vary in HIV-infected patients, emphasizing the need for optimized strategies. Therapeutic HPV vaccines in HIV-positive individuals show promising outcomes, and prophylactic HPV vaccines are considered safe and effective, with potential benefits for older individuals, necessitating further research in this age group. Physicians should prioritize vaccinations in HIV-positive individuals as part of comprehensive healthcare management. Tailored vaccination strategies, including vaccine selection, timing, and monitoring, can help optimize immune responses and improve overall health outcomes in this population.

Physicians should consider individual patient factors such as age, HIV status, CD4+ cell counts, and viral load suppression when recommending vaccinations. Vaccination timing should be considered once virologic suppression has been achieved to maximize immune response. Further research is needed to better understand the effectiveness and safety of vaccinations in HIV-positive individuals, particularly in older age groups.

## Figures and Tables

**Table 1:** Comparison of vaccines recommended in HIV-positive individuals.

Vaccine	Formulation	Immunogenicity	Side Effects	Main References
Influenza	Inactivated	++	+	[16,17,18,19,20,21,22,23,24,25,26,27,28,29,30,31,32,33,34,35,36,37]
*S. pneumoniae*	Polysaccharide, protein conjugate	++	+	[38,39,40,41,42,43,44,45,46,47,48,49,50,51,52,53,54,55,56]
COVID-19	mRNA, viral vector	++	+	[57,58,59,60,61,62,63]
Hepatitis A	Inactivated	++	+	[64,65,66,67,68,69]
Hepatitis B	Recombinant	+	++	[70,71,72,73,74,75,76,77,78,79,80,81,82,83,84,85]
Human papilloma virus	Recombinant	++	+	[86,87,88,89]
